# The Effects of Antioxidants from Natural Products on Obesity, Dyslipidemia, Diabetes and Their Molecular Signaling Mechanism

**DOI:** 10.3390/ijms23042056

**Published:** 2022-02-12

**Authors:** Chindiana Khutami, Sri Adi Sumiwi, Nur Kusaira Khairul Ikram, Muchtaridi Muchtaridi

**Affiliations:** 1 Department of Pharmacology and Clinical Pharmacy, Faculty of Pharmacy, Universitas Padjadjaran, Jl. Raya Bandung-Sumedang KM 21, Sumedang 45363, Indonesia; chindiana19001@mail.unpad.ac.id (C.K.); sri.adi@unpad.ac.id (S.A.S.); 2 Institute of Biological Sciences, Faculty of Science, Universiti Malaya, Kuala Lumpur 50603, Malaysia; nkusaira@um.edu.my; 3 Centre for Research in Biotechnology for Agriculture (CEBAR), Kuala Lumpur 50603, Malaysia; 4 Department of Pharmaceutical Analysis and Medicinal Chemistry, Faculty of Pharmacy, Universitas Padjadjaran, Jl. Raya Bandung-Sumedang KM 21, Sumedang 45363, Indonesia

**Keywords:** obesity, oxidative stress, dyslipidemia, diabetes, antioxidant

## Abstract

Obesity is a risk factor that leads to the development of other diseases such as dyslipidemia and diabetes. These three metabolic disorders can occur simultaneously, hence, the treatment requires many drugs. Antioxidant compounds have been reported to have activities against obesity, dyslipidemia and diabetes via several mechanisms. This review aims to discuss the antioxidant compounds that have activity against obesity, dyslipidemia and diabetes together with their molecular signaling mechanism. The literature discussed in this review was obtained from the PUBMED database. Based on the collection of literature obtained, antioxidant compounds having activity against the three disorders (obesity, dyslipidemia and diabetes) were identified. The activity is supported by various molecular signaling pathways that are influenced by these antioxidant compounds, further study of which would be useful in predicting drug targets for a more optimal effect. This review provides insights on utilizing one of these antioxidant compounds as opposed to several drugs. It is hoped that in the future, the number of drugs in treating obesity, dyslipidemia and diabetes altogether can be minimized consequently reducing the risk of side effects.

## 1. Introduction

Obesity is a pathological condition of excessive fat accumulating in the tissues under the skin and spreading to the organs and tissues around the body. From a health perspective, obesity is malnutrition caused by long-term excessive consumption of unhealthy food. Obese patients have health problems, one of which is an increase in total cholesterol levels > 200 mg/dL. The World Health Organization (WHO) points out that obesity is a chronic disease and is one of the risk factors for degenerative diseases such as diabetes and dyslipidemia, as well as acute coronary disease, hypertension, hyperuricemia and polycystic ovary syndrome [1]. Obesity can trigger oxidative stress through various mechanisms such as oxidative phosphorylation, glyceraldehyde autoxidation and superoxide formation [2]. Oxidative stress is a condition when there is an increase in the number of free radicals and/or a decrease in antioxidant activity [3]. Oxidative stress plays a role in comorbid obesity such as diabetes, dyslipidemia, endothelial dysfunction and mitochondrial dysfunction [2].

Patients with obesity are often associated with lipid abnormalities [4]. Approximately 60–70% of obese patients have dyslipidemia. In addition, insulin resistance disorders also contribute to the development of dyslipidemia. In recent years, dyslipidemia caused by the combined action of insulin resistance and obesity has been referred to as “metabolic dyslipidemia” with the main characteristics of increasing levels of triglycerides (TG) and decreasing levels of high-density lipoprotein (HDL). Under this condition, there can also be an increase in low-density lipoprotein (LDL) levels [5].

Diabetes mellitus (DM) is one of the comorbidities of obesity caused by oxidative stress. DM is a chronic disease affecting the body’s metabolism, characterized by increased blood sugar levels exceeding normal limits. In Southeast Asia, the number of diabetes cases in 2019 reached 88 million people and 90% of them were type 2 diabetes mellitus, half of which have complications that lead to death. The International Diabetes Federation (IDF) listed Indonesia as the 7th highest diabetic country with a prevalence of 8.5 million, and the number is predicted to increase to 14.1 million by 2035 [6]. The study by Mohieldein et al. (2015) reported that prediabetes was associated with obesity, development of dyslipidemia and decreasing total antioxidant status. Lifestyle changes such as weight loss, regular physical activity and a healthy diet should be encouraged to prevent progression to type 2 diabetes and its complications from prediabetes [7].

The hyperglycemia condition in DM has a significant impact on the vascular endothelium, which is caused by the auto-oxidation of glucose during the formation of free radicals, which in turn leads to macro- and microvascular dysfunction due to oxidative stress [3]. Oxidative stress conditions in DM are usually associated with an increase in endothelial cell apoptosis, which shows an increase in the free radical formation and a decrease in antioxidant capacity [8].

Metabolic disorders such as obesity, dyslipidemia and diabetes are the main causes of life-threatening ischemic heart disease [9]. Based on research by Vona et al. (2019) and Pechánová et al. (2015), it is reported that this metabolic disorder is accompanied by chronic inflammation mediated by oxidative stress. Increased oxidative stress in metabolic disorders plays a role in causing mitochondrial dysfunction, accumulation of protein and lipid oxidation products and disruption of the antioxidant system [10,11]. Clinical studies show that obesity co-occurring with metabolic disorders such as dyslipidemia and diabetes will increase the risk of death compared to obesity without metabolic disorders. However, when compared with lean individuals, obesity may increase the risk of death from various complications that accompany this condition [12].

At present, many people have adopted a healthy lifestyle, such as eating foods or taking medications derived from natural ingredients, especially those containing antioxidant compounds that can prevent and treat various diseases [13]. Compounds with antioxidant activity such as kahweol have been reported to have antidiabetic properties by suppressing pancreatic cell apoptosis and increasing insulin secretion in streptozotocin (STZ)-induced mice [14]. Another study by Pan et al. (2018) reported that flavonoids such as resveratrol (3,4′,5-trihydroxy-stilbene, RES) are widely present in vegetables and fruits with biological and pharmacological effects such as antiobesity, antioxidation activity and antidiabetic [15]. The combination of resveratrol and quercetin has also been reported to reduce hyperglycemia, serum glucose dysfunction and dyslipidemia in streptozotocin (STZ)-induced diabetic rats [16].

Of the many studies that discuss metabolic disorders and antioxidants from the literature, only two studies are obtained, which are closest to the discussion in this review. The two studies are conducted by Dal et al. (2016) and Shabbir et al. (2021). In a review article by Dal et al. (2016), the effect of consuming antioxidants from various sources such as functional foods, plants, fruits, vegetables, vitamins, supplements and other natural sources rich in polyphenols on diabetes and vascular complications based on in vivo, in vitro and clinical trials in humans were discussed [17]. Another review article by Shabbir et al. (2021) discusses the activity of polyphenol antioxidant compounds, namely, curcumin, quercetin and catechins, against metabolic disorders that focus on the role of the gut microbiota, which is affected by these antioxidant compounds to improve metabolic disorders [18].

In the two aforementioned reviews, there has been no detailed discussion on the effect of antioxidant compounds and their molecular signaling mechanisms against obesity, dyslipidemia and diabetes. The novelty of this review is the summary of information on antioxidant compounds derived from natural products based on the results of in vivo, in vitro and clinical trials that can treat obesity, dyslipidemia and diabetes. This could provide an insight on the antioxidant compounds that can simultaneously act as an antiobesity, antidyslipidemia and antidiabetic as compared to the current practices that require several drugs for the treatment of the three metabolic disorders, hence minimizing the use of multiple drugs and the risk of side effects and drug interactions. Identifying the plants containing these valuable compounds could also potentially yield a cheaper alternative treatment in the form of a herbal preparation to treat all three illnesses in the future.

In addition, this review also provides information on the molecular signaling pathways influenced by the antioxidant compounds from natural products that play a role in the development of obesity, dyslipidemia and diabetes. This would be useful for researchers to further investigate the activity of these antioxidant compounds to determine therapeutic targets.

## 2. Method

This review was made based on the results of the collection and review of journals obtained from the PUBMED database with several related keywords such as “antioxidant AND natural product AND obesity”, “antioxidant AND natural product AND antidiabetes”, “antioxidant AND natural product AND antidyslipidemia”, “signaling pathways AND natural product”, “obesity AND dyslipidemia AND natural product”, “antioxidant AND oxidative stress AND obesity AND dyslipidemia AND diabetes”, “ resveratrol AND metabolis disorders AND clinical study”, “quercetin AND clinical study AND antiobesity”, “curcumin AND antiobesity AND clinical study”, ”anthocyanins AND clinical study AND anti obesity”, “antioxidant AND metabolic disorders AND clinical study”, “antioxidant AND antiobesity AND antidiabetes”, “antioxidant AND antidislipidemia AND antidiabetes”.

The inclusion criteria for the main article are articles published in ≥2016 and research articles that discuss pharmacological antioxidant activity against obesity, dyslipidemia and diabetes as well as the signaling pathways of these antioxidant activities. Inclusion criteria for supporting articles are articles that discuss the metabolic disorders of obesity, dyslipidemia, diabetes and oxidative stress including the mechanism of metabolic disorders and the relationship between these metabolic disorders. This supporting article is taken from articles published between 2000 and 2021 with most of the articles included being published after 2016. Exclusion criteria for the main articles were duplicate articles, review articles, research with crude extracts and unrelated articles/irrelevant articles that do not discuss in detail the activity of the chemical compounds contained.

**Figure 1:**
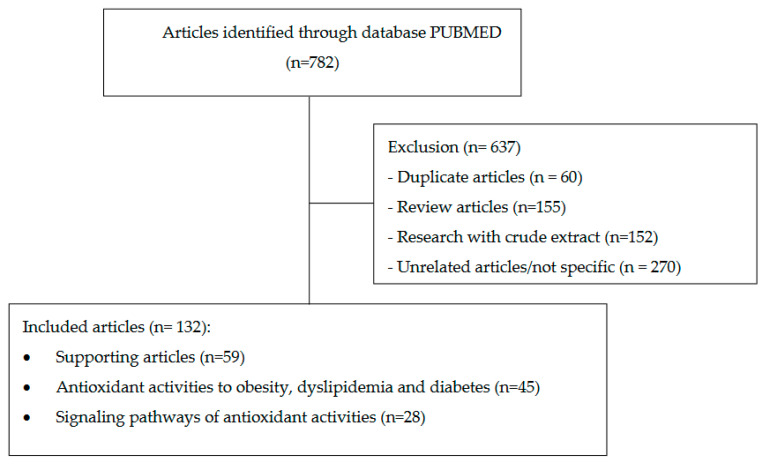
Article literature search flow chart.

Based on the search results using related keywords, 782 journals were obtained, which were then reduced after removing 60 duplicate articles, 155 review articles, 152 research articles with crude extracts and 270 unrelated/not specific articles. The results are 145 selected articles consisting of 49 supporting articles, 68 articles discussing antioxidant activity against obesity, dyslipidemia and diabetes and 28 articles discussing signaling pathways of antioxidant activity, which were used to be studied in this review. The article search flow can be seen in Figure 1.

## 3. Oxidative Stress and Its Relation to Metabolic Disorders (Obesity, Dyslipidemia and Diabetes)

Oxidative stress is a condition of an imbalance of production and accumulation of reactive oxygen species (ROS) in cells and tissues with the ability of biological systems to detoxify these reactive products [19]. Reactive oxygen species (ROS) in normal amounts contribute to various physiological processes such as hormone biosynthesis, defense systems, cellular signaling and fertilization. However, increased ROS production results in a condition known as oxidative stress, which has implications for various diseases such as diabetes, dyslipidemia, hypertension, atherosclerosis, heart failure, stroke and other chronic diseases [20,21]. Oxidative stress contributes to the development of obesity’s comorbidities [2]. Possible contributors to oxidative stress in obesity include increased hyperglycemia in tissue, lipids, vitamin and mineral deficiencies, chronic inflammation, endothelial dysfunction and impaired mitochondrial function [19].

### 3.1. Obesity

Obesity is one of the metabolic disorders resulting from an imbalance between energy intake and consumption. Obesity is marked by the inflammation of many cells, including macrophages [22] and adipose tissue [23]. Macrophages produce cytokines such as IL-6 and tumor necrosis factor alpha (TNFα), which also play a role in causing insulin resistance. In addition, obesity can cause the body’s resistance to insulin, mediated in part by free fatty acids (FFA) and adipokines such as retinol binding protein-4 (RBP4) and resistin, which can reduce insulin sensitivity [24]. Obesity is the main cause of metabolic syndromes such as type 2 diabetes mellitus, insulin resistance, dyslipidemia, hypertension and non-alcoholic fatty liver diseases (NAFLD) [1,25].

Obesity is known to have a relationship with the incidence of oxidative stress. Based on research done on several obese patients, it was reported that abdominal obesity may affect the occurrence of inflammation that triggers an increase in oxidative stress [26]. Studies show that obesity in visceral adipose tissue contributes to a state of oxidative stress that may lead to insulin resistance [27,28].

### 3.2. Dyslipidemia

Dyslipidemia is a serious problem because it is a major risk for coronary heart disease. Dyslipidemia is caused by several factors such as genes, diet, lifestyle, obesity, and many more [29]. Dyslipidemia in obesity consists of elevated levels of triglycerides (TG) and free fatty acids (FFA), decreased and dysfunctional high-density lipoprotein (HDL) and a slight increase of low-density lipoprotein-cholesterol (LDL) levels. In addition, the concentration of apolipoprotein B (apo B) may also increase due to excessive production of apo B in the liver containing lipoproteins [30].

Dyslipidemia in obesity is characterized by the occurrence of hypertriglyceridemia due to the accumulation of triglycerides in the liver. This leads to the inhibition of chylomicron lipolysis caused by increased synthesis of very low-density lipoprotein (VLDL) in the liver due to the competition for lipoprotein lipase (LPL). Hypertriglyceridemia will induce an increase in the exchange of cholesterol esters (CE) and triglycerides between VLDL, LDL and HDL via cholesteryl ester transfer protein (CETP) [25].

Based on previous research, hypercholesterolemia may induce apoptosis and autophagy caused by ROS activation [31]. Increased production of ROS affects the development of dyslipidemia and other cardiovascular diseases. Free radicals function physiologically as signal transducers and maintain homeostasis in cellular signaling, but when these pathways are disrupted, they may cause risk factors for atherosclerosis, one of which is dyslipidemia [32,33].

### 3.3. Diabetes Mellitus

Diabetes mellitus (DM) is a metabolic disorder characterized by increased blood sugar levels. DM can be divided into two types: DM type 1 and DM type 2. DM type 1 is caused by the unavailability of insulin produced by pancreatic beta cells, which can be caused by genetic or autoimmune disorders, while type 2 DM occurs due to lack of insulin secretion or insulin resistance, or both. DM type 2 can occur due to the influence of genetic, epigenetic or lifestyle factors [34].

Various inflammatory cytokines such as IL-1β produced by M1 macrophages can cause local and systemic inflammation, pancreatic cell dysfunction and insulin resistance in the liver, adipose and musculoskeletal tissues [35]. M1 macrophages are also associated with diabetes complications, such as kidney disease, neurological diseases, retinopathy, and cardiovascular diseases. However, to date, the underlying mechanism of M1 macrophage accumulation in diabetic patients is not fully known [22].

Hyperglycemia in prediabetes may trigger oxidative stress and increase inflammatory factors that affect vascular dysfunction. This oxidative stress may also cause interference with glucose uptake from muscle cells and fat cells and may reduce insulin sensitivity [27]. Another study also reported an increase in ROS concomitantly with suppression of the antioxidant enzyme superoxide dismutase (SOD) in rats induced by hyperglycemia [36].

## 4. Antioxidant Activities from Natural Products to Treat Obesity, Dyslipidemia and Diabetes Mellitus

Antioxidants are substances that can counteract free radicals and prevent free radicals from damaging cells. Free radicals are the root cause of health problems, such as cancer, premature aging, cardiovascular disease and digestive diseases. The body naturally produces antioxidants, but when free radicals are abundant, this process will not be efficient and its effectiveness also decreases with age. Increasing the intake of antioxidants can prevent various diseases and reduce health problems. Food such as fruits and vegetables contain important antioxidants such as vitamins A, C, E and beta-carotene, as well as essential minerals such as selenium and zinc [37]. Several antioxidant compounds from natural ingredients that have been widely studied and have antiobesity, antidyslipidemia and antidiabetic activity are resveratrol, curcumin, quercetin and anthocyanin as well as other antioxidants.

### 4.1. Resveratrol

Resveratrol (3,5,4′-trihydroxy-trans-stilbene) is a natural antioxidant compound that can be found in various plants, such as *Polygonum cuspidatum*, peanuts and fruits, such as grapes and berries. The use of resveratrol as a nutraceutical has also been widely reported based on testing in animal and human models as a treatment for obesity, metabolic disorders and cardiovascular disorders [38]. Resveratrol is a polyphenolic antioxidant compound that has various biological activities and has been used as a dietary supplement [38].

A clinical study was conducted on 13 patients with type 1 diabetes who were given resveratrol in 500 mg capsules for 60 days. From the results of this study, it is known that the administration of resveratrol can reduce fasting blood sugar (FBS) significantly (*p* < 0.05) compared to the initial value with FBS levels of 253.69 ± 49.67 vs. 174.38 ± 45.19 [39]. Another clinical study by Jorge et al. (2020) was conducted on 25 obese individuals (BMI 30 kg/m^2^) aged 30–60 years who were randomly assigned to a placebo group and a group given resveratrol at a dose of 250 mg/day accompanied by the same program of physical activity and diet carried out for three months. After 3 months, it was reported that in the resveratrol group there was a significant decrease (*p* < 0.05) in body weight, BMI, waist circumference, total cholesterol (TC), VLDL and a significant increase in HDL levels, while in the placebo group significant reduction in body weight, BMI and waist circumference was also reported but no significant reduction in lipid profile [40].

Another study by Rabbani et al. (2021) reported that administration of oral capsules containing a combination of trans-resveratrol and hesperetin (90 mg tRES: 120 mg HESP) for 8 weeks tested on obese patients showed a decrease in inflammation which was characterized by a decrease in the expression of IL-8 and receptor for the advanced glycation end product (RAGE). In addition, this combination also improves insulin resistance and hyperglycemia [41].

In vivo testing has been carried out on rats induced with a high-fat diet for 12 weeks which were then given resveratrol at a dose of 20 mg/kg/day for 4 weeks, and the results showed a reduction in total cholesterol by 8.4% and LDL by 6.6%. compared to the HFD group (hyperlipidemia group) [42]. Another study was conducted by Campbell et al. (2019) on male C57BL/6J rats which were given a high-fat diet for 16 weeks accompanied by the administration of resveratrol doses of 50, 75 and 100 mg/kg body weight via drinking water. Based on the results, the administration of resveratrol doses of 75 and 100 mg/kgbw could significantly (*p* < 0.05) prevent weight gain in rats compared to the HFD group. In addition, the administration of resveratrol doses of 75 and 100 mg/kgbw was also reported to prevent chronic inflammation, which was characterized by a decrease in serum IL-1 and TNFα (*p* < 0.05), as well as oxidative stress in the liver and brain as indicated by an increase in activity of superoxide dismutase, catalase and glutathione peroxidase (*p* < 0.05) [43].

Research on the antiobesity activity of resveratrol compounds has also been carried out by Chang et al. (2016) in vivo and in vitro. In vivo testing activity of resveratrol was carried out on male C57BL/6C rats induced with high fat diet (HFD) accompanied by the administration of resveratrol at doses of 1, 10 and 30 mg/kgbw for 10 weeks with the results showing that the administration of resveratrol with these three doses may significantly attenuate dose-dependent HFD-induced weight gain compared to the HFD group without any treatment. Furthermore, in vitro testing on 3T3-L1 cells by administering resveratrol at a concentration of 0.03 to 100 μM for 24 h significantly inhibited dose-dependent adipose lipolysis [44]. Based on in vivo, in vitro and clinical trials discussed previously, resveratrol is a potent antioxidant compound in overcoming metabolic disorders of obesity, dyslipidemia and diabetes. However, there is very little information about the dosage and safety for long-term use of this compound, hence, further research is needed to provide an optimal effect of resveratrol and reduce the risk of side effects.

### 4.2. Curcumin

Curcumin found in turmeric root (*Curcuma longa* L.) has been reported to have physiological effects such as antioxidant, antiobesity, anti-inflammatory, antidyslipidemic and antidiabetic. Turmeric is widely consumed in Asian countries and used as a cooking spice with no reported toxicity [45]. In vitro assays were carried out by Zhao et al. (2021) on 3T3-L1 preadipocytes and the results showed that incubation of curcumin at doses of 10, 20 and 35 μM for 8 days could induce adipogenic differentiation and accumulation of intracellular fat droplets. These results also showed that there was a 55.0% and 74.7% decrease in preadipocyte viability compared to the control group on incubation with 50 μM and 75 μM curcumin, respectively (*p* < 0.01). Administration of curcumin has also been reported to enhance mitochondrial respiratory function, induce adipogenic differentiation and regulate peroxisome proliferation activated receptor γ (PPARγ) and peroxisome proliferator-activated receptor gamma coactivator 1-alpha (PGC1α) expression [46,47].

In vivo testing was carried out on male albino wistar rats induced with HFD for 4 weeks followed by administration of curcumin at a dose of 80 mg/kg body weight/day for the next 6 weeks. The results showed that there was a significant decrease in BMI (*p* < 0.05) in rats given curcumin, a BMI value of 0.78 g/cm^2^ compared to the obese group with a BMI of 0.86 g/cm^2^ [48].

Clinical trials have been carried out by Thota et al. (2019) on several individuals with a high risk of developing diabetes, having a BMI ranging from 25–45 kg/m^2^, with fasting glucose levels of 6.1–6.9 mmol/L and HbA1c levels between 5.7–6.4% were given curcumin tablets at a dose of 2 × 500 mg curcumin/day taken every morning and night for 12 weeks. The results of this study indicate that curcumin can increase insulin sensitivity by 32.7 ± 10.3%, reduce serum triglycerides by 0.79%, compared to the placebo group, which had an increase of 26.89%, and significantly reduce insulin resistance (−0.3 ± 0.1 vs. 0.01 ± 0.05, *p* = 0.0142), as compared to the placebo group [49,50].

In vivo testing was carried out on male wistar rats by inducing rats with an intraperitoneal injection of nicotinamide (110 mg/kg) and streptozotocin (45 mg/kg) in a fasting state. The results of this study by Goushki et al. (2020) reported that the administration of curcumin (100 and 200 mg/kg/day) and nano curcumin (100 and 200 mg/kg/day) could significantly (*p* < 0.001) reduce fasting blood sugar (FBS) with FBS levels respectively also 158.13, 163.75, 173.38 and 158 mg/dl, compared to the FBS value in the diabetes group of 518.5 mg/dl [51]. In addition, a study by Roxo et al. (2019) was also carried out on male wistar rats induced with STZ at a dose of 40 mg/kg IV so that diabetic rats with blood sugar levels in the range of 380–510 mg/dl were then given a dose of curcumin, 30 mg/kg, 60 mg/kg and 90 mg/kg, and the results showed that none of these doses caused toxicity in rats. Furthermore, the study reported that administration of curcumin at a dose of 90 mg/kg could improve the lipid profile, which was indicated by a significant decrease (*p* < 0.05) in plasma triacylglycerol and cholesterol levels compared to diabetes controls, and inhibit the advanced glycation end products (AGE)/RAGE signaling pathway [52,53].

Tests in streptozotocin-induced diabetic mice show that tetrahydrocurcumin (THC) at a dose of 120 mg/kg/day for 12 weeks can relieve diabetic cardiomyopathy by attenuating oxidative stress due to hyperglycemia and activating the SIRT1 pathway [54]. Other tests by Lima et al. (2020) reported that administration of curcumin at a dose of 90 mg/kg for 45 days can significantly increase the activity of antioxidant enzymes such as superoxide dismutase, paraoxonase 1 and catalase in 40 mg/kg STZ-induced rats compared to negative controls [55].

Based on a study by Li et al. (2019), the administration of curcumin 20 μM for 24 h at 37 °C to INS-1 cells induced with high glucose/palmitate could effectively inhibit oxidative stress, cell proliferation, increase insulin levels and reduce nicotinamide adenine dinucleotide phosphate (NADPH) oxidase expression as compared to high palmitate (PH) [56], while an in vivo study conducted on C57BL/6J rats induced with a high-fat diet for 3 months and then given curcumin at a dose of 1.5 g/kg/day for 8 weeks showed that curcumin can protect islet cells of Langerhans from apoptosis by modulating the NADPH pathway [57].

Based on the results of curcumin testing in vivo, in vitro and based on clinical trials, it is known that curcumin compounds have good potential in the treatment of metabolic disorders of obesity, dyslipidemia and diabetes. However, further research is needed to obtain the optimal dose, the appropriate dosage form, safety testing for long-term use and the possible side effects so that it can provide optimal effects on metabolic disorders of obesity, dyslipidemia and diabetes in humans.

### 4.3. Quercetin

Quercetin belongs to the class of flavonols found in many fruits and vegetables such as apples, berries, cauliflower, cabbage and beans. Quercetin has also been widely studied as having antioxidant, antidyslipidemic, antidiabetic, anti-inflammatory and other activities [58]. In vitro tests were carried out on 3T3-L1 adipocytes, and it was reported that administration of pentamethylquercetin (PMQ) at concentrations of 1 and 10 M increased glucose consumption by 24.6% and 66.4% (*p* < 0.05 and *p* < 0.01 vs. vehicle). This suggests that PMQ can increase insulin activity in 3T3L1 adiposity. Furthermore, in vivo testing was carried out on wistar rats induced with HFD and given PMQ at a dose of 0.04% g/g for 17 weeks. The test results showed a significant decrease (*p* < 0.05) in serum glucose, TC, TG and LDL levels in rats given PMQ compared to the HFD group of rats [59]. Tests on rats induced using STZ reported that there was a significant decrease (*p* < 0.05) in body weight in diabetic rats, and administration of 75 mg/kg bw of quercetin for 28 days showed an increase in body weight of 17.83%, a decrease in blood glucose of 66.80%, triglycerides of 24%, VLDL of 48%, LDL of 31.75% and total cholesterol 26.43% and an increase in HDL of 78.88%, compared to the group of HFD mice [60].

In vivo testing was carried out on wistar rats with STZ induction of 55 mg/kg bw, and an excision wound of 2 cm × 2 cm (400 mm^2^) was made. Then, after being declared diabetic on blood sugar measurements after 72 h, quercetin was given orally at a dose of 100 mg/kg body weight + quercetin ointment (1%) for 21 days. The results showed that quercetin can normalize changes in blood glucose levels comparable to the normal control group with blood sugar levels ranging from ±150 mg/dl, while the blood sugar levels of the HFD group were ±350 mg/dl and could heal wound areas in diabetic rats significantly greater than the diabetes group [58]. A study by Zhuang et al. (2018) reported that administration of quercetin isolated from *Edgeworthia gardneri* at a dose of 0.5 g/kg quercetin per day for 4 weeks in db/db mice with type 2 diabetes mellitus (T2DM) could induce insulin secretion at a concentration of 0.10 mol/L, inhibit palmitate-induced pancreatic cell apoptosis and ameliorate mitochondrial dysfunction [61].

Clinical testing was conducted by Lee et al. (2016) on obese male and female Korean individuals who were given quercetin-rich onion peel extract (OPE) capsules at a dose of 100 mg for 12 weeks. The results obtained were that quercetin-rich OPE supplementation significantly reduced body weight and BMI from 70.0 ± 11.4 to 69.2 ± 11.4 kg (*p* = 0.02) and BMI from 26.6 ± 3.3 to 26.3 ± 3.2 kg/m^2^ (*p* = 0.03), whereas in the placebo group there was no significant change. Waist and hip circumference showed significant changes in both groups. The waist circumference of the control group decreased from 90.2 ± 6.5 to 89.5 ± 6.4 cm, while the OPE group decreased by 2 cm from 91.9 ± 7.6 to 89.9 ± 7.7 cm. The hip circumference of the control group decreased from 100.7 ± 5.2 to 99.9 ± 4.6 cm, while the OPE group decreased by 1.3 cm from 101.1 ± 5.9 cm before the experiment to 99.9 ± 6.3 cm after the experiment. In addition, skinfold thickness in the control group decreased significantly by 2.2 mm from 33.2 ± 5.5 to 31.1 ± 5.6 mm (*p* < 0.001), whereas in the OPE group it decreased significantly by 3.2 mm from 34.1 ± 7.1 to 30.9 ± 6.4 mm (*p* < 0.001). OPE also showed a significant reduction in arm fat percentage by 0.7% from 36.1% ± 8.8% to 35.5% ± 5.5% (*p* = 0.03) and total body fat by 0.6% from 38.2% ± 6.5% to 37.6% ± 6.4% (*p* = 0.02) [62].

### 4.4. Anthocyanin

Anthocyanins are polyphenolic compounds found in pigmented fruits and vegetables. It is reported that this compound has pharmacological activities such as antioxidant, anti-inflammatory and anti-obesity [63]. In vitro assays were carried out on 3T3-L1 cells by administering 5, 10, 15, 20, 25, 30, 50, 100 and 200 g/mL anthocyanin fraction (AnT Fr) for 24 h, and the results showed the inhibition of lipid accumulation via regulation of adipogenesis and lipogenesis-related genes and signaling proteins [64]. An in vitro study by Han et al. (2018) reported that administration of anthocyanins at a dose of 200 g/mL showed a lipid reduction of 60% [65] and the administration of 10 g/mL can decrease ROS and increase catalase (CAT) and superoxide dismutase (SOD) enzymes significantly [66]. A study by Suantawee et al. (2017) reported that administration of cyanidin, an anthocyanin, at a dose of 1–300 μM can increase insulin release from INS-1 cells and stimulate insulin secretion [67], whereas administration at 60, 100 and 300 μM increased insulin secretion six times higher than the control [68]. In addition, in vivo studies on obese rats reported that administration of blackberry anthocyanins (BLA) and blueberry anthocyanins (BBA) at a dose of 200 mg/kg food for 12 weeks could inhibit body weight gain by 40.5% and 55.4%, respectively [63].

A clinical trial was conducted by Zhang et al. (2020) on dyslipidemic patients who were given anthocyanins in the form of supplements to see the dose-response relationship of oxidative stress and inflammation in dyslipidemic patients. Based on these tests, it was found that anthocyanin supplementation (320 mg/day) for 6 weeks significantly increased total-SOD compared to the placebo (*p* < 0.05). Anthocyanins (80 mg/day) significantly reduced serum IL-6 (−20%), TNF-α (−11%) and urinary 8-iso-PGF2α (−27%) versus the placebo (*p* < 0.05). A dosage of 320 mg/day anthocyanin supplementation can significantly reduce serum IL-6 (−40%), TNF-α (−21%) and malondialdehyde (MDA) (−20%) [69].

### 4.5. Other Antioxidants

Obesity may cause a decrease in total antioxidant capacity (TAC) in obese compared to normal individuals. This condition may also lead to a decrease in HDL levels [70]. Various in vivo and in vitro studies have been carried out to investigate the activity of antioxidant compounds from natural ingredients towards obesity. Lemon is known to have antioxidant activity, and lemon fermented product (LFP) at 0.75 and 1 mg/mL for 10 days was reported to inhibit the accumulation of lipids by 8.3% in 3T3L1 adipocytes. In addition, based on in vivo studies using mice, LFP at a dose of 2.89 g/kg for 9 weeks can reduce the body weight of obese mice by 9.7%, decrease triglyceride levels (17.0%), glucose (29.3%) and free fatty acids (17.9%) and can increase serum HDL (17.6%) [71].

In other studies, by Liao et al. (2019), antioxidant polysaccharides okra (OP) derived from okra (*Abelmoschus esculentus* L.) at doses of 200 and 400 mg/kgbw showed a significant reduction of dyslipidemia in rats induced by a high-fat diet and streptozotocin 100 mg/kg. The lipid profiles such as total cholesterol, triglycerides and LDL were significantly reduced as compared to the negative control group. These studies also reported that there was an increase in antioxidant enzymes at a dose of 400 mg/kg of OP such as superoxide dismutase (sod), catalase (cat) and glutathione peroxidase (gsh-px) by 274.18 ± 24.1, 57.09 ± 6.91 and 530.08 ± 45.1 u/mg prot respectively [72].

Clinical trials were conducted on healthy individuals aged 30–75 years who consumed green tea in combination with glucosyl hesperidin (GT gH), which contained 178 mg glucosyl hesperidin and 146 mg epigallocatechin gallate (EGCG), for 12 weeks. The results showed that GT gH prevented the addition of body weight, and the antiobesity effect of GT gH is more pronounced in people < 50 years old [73].

In vivo testing was carried out on wistar rats fed with strawberry ellagitannins (ET), which showed that a level of 0.24% of the total diet for 4 weeks can be used effectively for the prevention and treatment of metabolic disorders associated with obesity, dyslipidemia, imbalanced redox status and inflammation [74]. In vivo testing was also carried out by Sousa et al. (2020) on male Sprague-Dawley rats induced on a high-fat diet then given α-terpineol at a dose of 50 mg/kg, which improved insulin sensitivity and reduced (*p* < 0.05) serum levels of the proinflammatory cytokines TNF-α and IL-1β, when compared with the control group [75].

## 5. Effect of Antioxidants on Metabolic Disorders of Obesity, Dyslipidemia and Diabetes

### 5.1. Relationship between Obesity, Dyslipidemia and Diabetes

In the obese population, there is a decrease in skeletal muscle strength and function as well as impaired skeletal muscle mitochondrial respiratory function that contributes to increased mitochondrial ROS production compared to normal-weight individuals [76]. It has been reported that the ratio of type II and type I skeletal muscle fibers is higher than that of normal individuals, with two to three-fold ROS production [77]. Tumor necrosis factor alpha (TNF-α) functions as a catalyst in oxidative stress which is only expressed by type II muscle fibers. Based on studies, systemic administration of TNF-α has been shown to reduce the production of skeletal muscle strength in test animals and can increase muscle protein loss through oxidative activation of the TNF-α/nuclear factor kappa B (NF-κB) signaling pathway. Skeletal muscle oxidative stress induced by TNF-α is preventable by the administration of antioxidants, suggesting that TNF-α may provide an important target for confirming obesity-associated oxidative stress [78].

**Figure 2:**
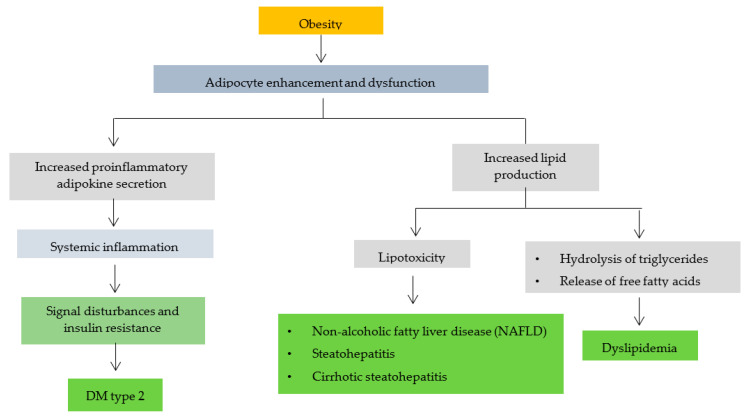
The relationship of obesity with other disease complications (modification of B. Heymsfield et al., 2017) [81].

Obesity can trigger various complications, as shown in Figure 2. Obesity leads to increased and dysfunctional adipocytes [79]. Adipocytes produce adipokines and hormones whose rate and effect of secretion are influenced by the distribution and amount of available adipose tissue. High secretion of pro-inflammatory adipokines by adipocytes and macrophages may cause systemic inflammation in some obese patients [80]. The accumulation of excess lipid intermediates (such as ceramides) triggers lipotoxicity with cell dysfunction and apoptosis in some non-obese tissues. Inflammatory cytokines that are elevated in non-adipose tissue cause impaired signaling and insulin resistance, especially in obese patients, leading to type 2 DM [81].

Increased adipocytes can also trigger increased lipid production, causing the triglycerides in adipocytes to hydrolyze and release free fatty acids (FFA). Dilation of adipose tissue causes high plasma FFA levels in some patients. Apart from adipose tissue, lipids can also be found in liposomes [82]. Too many fat cells can cause liposomes (steatosis) in liver cells to expand and form large vacuoles associated with diseases, including non-alcoholic fatty liver disease (NAFLD), steatohepatitis and cirrhotic steatohepatitis [83]. This finding is one of many pathophysiological mechanisms of obesity-induced dyslipidemia (increased triglyceride levels, LDL, decreased HDL), type 2 DM, obesity-associated liver disease and osteoarthritis [84,85,86].

In obese patients, there is an increase in reactive oxygen species (ROS) and a decrease in antioxidant defense. Increased oxidative stress in obesity can cause inflammation. In addition, the hormone leptin secreted by adipocytes also plays a role in inducing oxidative stress [87]. Oxidative stress plays a role in causing insulin resistance leading to type 2 diabetes and dyslipidemia [88].

Compounds with antioxidant activity in the treatment of type 2 diabetes can activate the 5′adenosine monophosphate-activated protein kinase (AMPK) pathways, down-regulate the expression of cyclooxygenase-2 (COX2) related genes to release pro-inflammatory mediators, increase glucose tolerance and insulin sensitivity, reduce inflammatory cells and reduce cytokines levels. Pro-inflammatory agents in the serum, such as IL-1B, IL-6 and TNF-α, can inhibit the activation of NF-κB and inhibit the expression of macrophage chemotactic protein (MCP1) [89]. It is reported that the use of antioxidants in patients with type 2 diabetes can effectively prevent complications, which is supported by various studies on antioxidants and the pathological process of diabetes caused by increased oxidative stress [13].

### 5.2. Antioxidant Mechanisms Associated with Obesity, Dyslipidemia and Diabetes

Several antioxidant compounds have been studied and have activity against obesity, oxidative stress, dyslipidemia and diabetes. Kukoamine B compounds are known to have activity in preventing inflammation and reducing lipid accumulation and oxidative stress [90]. Another antioxidant compound, salidroside, has also been investigated to have activity in inhibiting the formation of ROS that leads to oxidative stress [91,92]. In addition, salidroside can also protect cells from apoptosis caused by H_2_O_2_ induction [93]_._ The antioxidant compound polysaccharide okra (OP) is known to prevent an increase in levels of free fatty acids (FFA), triglycerides and LDL and can prevent a decrease in HDL levels [72].

Kahweol is an antioxidant diterpene compound derived from coffee. Based on research, kahweol can inhibit adipogenesis and lipid accumulation while lowering blood glucose levels in rats induced by hyperglycemia [14,94]. Besides playing a role in inhibiting ROS and suppressing lipid accumulation, antioxidant compounds also have activity against insulin resistance. An antioxidant compound known to affect insulin resistance is asphalathin. Asphalathin can improve insulin resistance in in vitro testing with palmitate induction [95,96], while other studies have also shown that asphalathin can treat hyperglycemia accompanied by inflammation and apoptosis [97].

**Figure 3:**
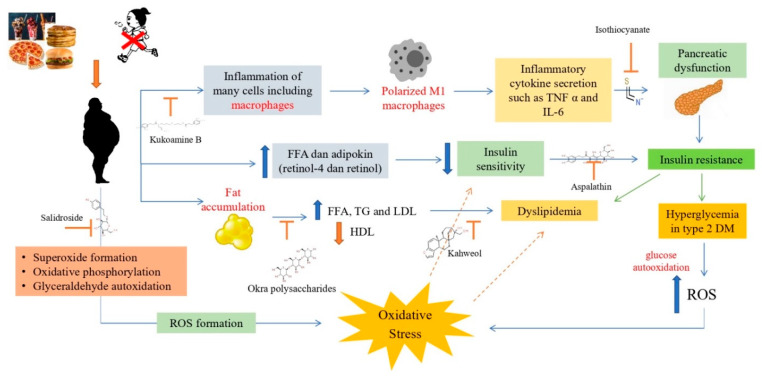
Antioxidant mechanisms in obesity, dyslipidemia and diabetes **[100]**. Obesity can cause inflammation of macrophage cells so macrophages will be polarized into M1 macrophages due to inflammation. M1 polarized macrophages will secrete inflammatory cytokines such as TNFα and IL-6, which can cause pancreatic dysfunction that leads to insulin resistance. Insulin resistance will cause type 2 DM with hyperglycemia, which can increase ROS, causing oxidative stress. Obesity can form ROS through the formation of superoxide, oxidative phosphorylation and auto-oxidation of glyceraldehyde, causing oxidative stress. Obesity can also cause an increase in FFA and adipokines, which can reduce insulin sensitivity and lead to type 2 diabetes and dyslipidemia. In addition, obesity also affects fat accumulation, which can cause an increase in FFA, TG and LDL and a decrease in HDL, which can cause dyslipidemia.

Another antioxidant compound that also affects insulin resistance is isothiocyanate. This compound reduced lipid accumulation and inflammation in the palmitate-induced test [98]. In another study, it was also reported that isothiocyanate compounds can suppress inflammation caused by an increase in pro-inflammatory cytokines and can reduce oxidative stress levels [99]. The activities of some of these antioxidant compounds against obesity, oxidative stress, dyslipidemia and diabetes are summarized in Figure 3.

## 6. Antioxidant Compound Signaling Pathways

Based on the previous discussion, it is known that several antioxidant compounds have antiobesity, antidyslipidemic and antidiabetic activities both in vitro and in vivo and clinically. Furthermore, this review discusses the signaling pathways of antioxidant compounds derived from natural products against obesity, dyslipidemia and diabetes as well as oxidative stress that can trigger the emergence of these metabolic disorders (Table 1).

### 6.1. The Phosphoinositide 3-kinase/Protein Kinase B (PI3K/AKT)

The phosphoinositide 3-kinase/protein kinase B (PI3K/AKT) signaling pathway is a regulator of physiological processes associated with type 2 diabetes mellitus. Most studies reported that the PI3K/AKT pathway not only promotes insulin signal transduction but can also stimulate glucose uptake in adipose and liver [72]. Phosphorylated protein kinases can activate glycogen synthase kinase 3 beta (GSK3β), which then triggers NF-E2-related factor (Nrf2) from the binding of Keap1 to the nucleus. Then, target genes are transactivated through antioxidant response elements (AREs) to inhibit oxidative stress. Some of the compounds in Table 1 that can induce vasodilation through the PI3K/AKT signaling pathway are polysaccharides [72], anthocyanin [101], resveratrol [120], gossypol [104], procyanidins [117] and polyphenol [114].

### 6.2. The Nuclear Factor Erythroid 2-Related Factor 2 (Nrf2) Signaling

The NFE2 system associated with kelch-like ECH-associated protein 1 (Keap1) factor 2 (Nrf2) is a defense system for cellular homeostasis. The interaction between Nrf2 and Keap1 can trigger the expression of the B globin gene known as a key marker of oxidative stress in cells [125]. Levels of oxidative stress and inflammation in cells are common in most tissues. Nrf2 and NF-κB (nuclear factor kappa-light-chain-enhancer of activated B cells) are the two main transcription factors that play a role in regulating cellular responses to oxidative stress and inflammation. There is functional crosstalk between these two pathways based on pharmacological and genetic studies which stated that NF-κB activity will be disrupted with the absence of Nrf2, causing an increase in cytokine production. In addition, NF-B also plays a role in modulating the activity and transcription of Nrf2 [126]. Based on Table 1, several natural compounds were reported to act on the Nrf2 cell homeostasis system such as isothiocyanates [99], ocra polysaccharides [72], simmondsine [123] and puerarine [118].

Moringa isothiocyanate (MIC1) is the main isothiocyanate found in *Moringa oleifera*. MIC-1 can activate Nrf2-ARE at levels similar to sulforaphane (SFN), suppress pro-inflammatory cytokines, reduce ROS and inhibit high glucose (HG)-induced transforming growth factor beta 1 (TGFβ1) [99]. Another antioxidant compound, namely, okra polysaccharide (OP), significantly reduces the increase in blood sugar, cholesterol, triglycerides and LDL. OP also decreases ROS and mitochondrial dysfunction by inhibiting activation of NADPH oksidase 2 (Nox2). In summary, OP has activity against type 2 DM via Nrf2 transport of the PI3K/AKT pathway [72]. Simmondsin and puerarin are also reported to reduce oxidative stress via the same mechanism, namely, by activating the Nrf2 pathway [118,123].

### 6.3. The Peroxisome Proliferation Activated Receptor γ (PPARγ)

The peroxisome proliferation activated receptor γ (PPARγ) is a transmembrane transcription factor. When activated by the ligand, PPARγ inhibits the transcription of NF-κB and reduces the expression of the cytokine gene in inflammation, which may decrease the inflammatory response. In vivo studies have shown that the induction of a high-fat diet can lead to an increase in glucose levels and insulin resistance accompanied by a decrease in PPAR activation [127]. Compounds reported in Table 1 with activity to increase PPARγ expression are kahweol [14], *Angelica sinensis* polysaccharide (ASP) [115] and toosendanin [128].

### 6.4. The Nuclear Factor Kappa-Light-Chain-Enhancer of Activated B Cells (NF-κB) Signaling

NF-κB is a transcription factor consisting of seven transcription factors that are structurally related and play a role in regulating the expression of many genes. NF-κB usually represents the p50-p65 heterodimer, which is the major Rel/NF-κB complex in most cells. The NF-κB subunit is expressed in many places but its induction and expression depend on the stimulus and cell type. NF-κB can be activated by cytokines, ROS, viral infection, vasopressors and DNA damage. NF-κB and Nrf2 play a role in cellular homeostasis and responses to stress and inflammation, whose molecular mechanisms depend on cell type and tissue context [129]. Based on Table 1, several natural compounds are reported to act on the NFκB cell homeostasis system such as kahweol [14], kukoamine B [106], saponins [122], oligopeptides [103] and hyperoside [105]. These compounds can prevent cell apoptosis due to the induction of hyperglycemia and downregulation of NFκB to protect against inflammation caused by diabetes [14,103].

### 6.5. 5′AMP-Activated Protein Kinase (AMPK) Signaling

**Figure 4:**
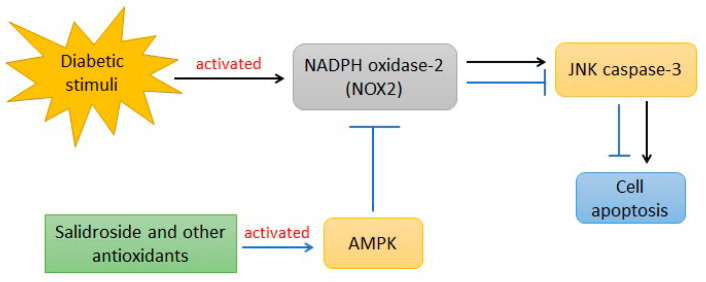
The salidroside mechanism in preventing oxidative diabetes (modification from Ju L et al., 2017) [121].

AMP-activated protein kinase (AMPK) plays a role in regulating energy metabolism through the inhibition of anabolic pathways and stimulation of catabolic pathways. In addition, AMPK also plays a role in enzyme regulation through phosphorylation and regulation of transcription factors and coactivators [130]. Based on Table 1, several natural compounds can increase AMPK levels, such as salidroside [121], aspalathin [102], nodakenine [110], anthocyanins [101], saponin [122], peptic bee pollen polysaccharide [112] and bouchardatine [102]. Salidroside (Figure 4) and other antioxidant compounds that act in the AMPK pathways can decrease ROS production, improve mitochondrial function by reducing NADPH oxidase-2 (NOX2) expression and inhibit the JNK-caspase 3 apoptotic cascade by activating AMPK [121]. 

### 6.6. AGE/RAGE

In conditions of persistent hyperglycemia of uncontrolled diabetes mellitus, the end product is advanced glycation end (AGE). Glycation is one of the mechanisms that contribute to diabetes complications such as cardiomyopathy, nephropathy, retinopathy and neuropathy [131]. Advanced glycation ends (AGEs) play a role in the disruption of cellular functions including denaturation of target proteins and reduced function of AGEs. The accumulation of AGEs in tissues can damage organs and trigger chemical oxidative stress. Another mechanism of AGEs is the receptor associated with the receptor for the advanced glycation end product (RAGE). The increase in RAGE causes an increase in ROS synthesis and oxidative stress. One of the oxidative stresses is the phosphorylation of the primary signal transduction cascade, which is the molecularly activated protein kinase (MAPK) that activates NF-κB [132]. In Table 1, the compounds with a AGE/RAGE inhibitory mechanism are mangiferin [108], morroniside [109] and pyrogallol-phloroglucinol-6,6-bieckol (PPB) [119].

### 6.7. SIRT (Sirtuin)

Sirtuin 1 (SIRT1) and sirtuin 3 (SIRT3) proteins play an important role in counteracting oxidative stress. In degenerative diseases such as type 2 diabetes, especially in women, there is a high risk of death from myocardial infarction, even with drug therapy for diabetes. Compounds that can increase SIRT1 are resveratrol and bouchardatine [102] (Table 1). Resveratrol (RSV) is a natural polyphenol that has antioxidant activity and can improve mitochondrial dysfunction. RSV has been shown to increase NO production, increase NOS expression and activity, prevent eNOS release and increase NO bioavailability [120].

## 7. Conclusions

Various antioxidant compounds have been reported to have beneficial activities against obesity, dyslipidemia and diabetes in the literature. The molecular signaling mechanism of the reported compounds associated with obesity, dyslipidemia and diabetes has also been discussed. However, further research is needed to determine the optimal dose of these antioxidant compounds so as to provide optimal effects on these metabolic disorders. Furthermore, the review also provides insights into antioxidant compounds that act simultaneously against obesity, dyslipidemia and diabetes to minimize the use of drugs and the risk of side effects. Further research on the side effects of long-term use of these antioxidant compounds should also be explored, thereby increasing the safety of long-term use of these compounds.

## Figures and Tables

**Table 1:** Antioxidant signaling pathways of natural product.

Compounds	Sources	Experimental Models	Mechanisms	Ref.
Anthocyanin (100 and 400 mg/kg for 5 weeks)	*Vaccinium corymbosum*	Streptozotocin-induced diabetic rats and HepG2 cells.	Hyperglycemia and hyperlipidemia are inhibited by reducing the expression of enzymes involved in gluconeogenesis, lipogenesis, and lipolysis via the adenosine monophosphate (AMPK)-activated kinase signaling pathway in HepG2 cells.	[101]
Aspalathin (10 g/mL for 3 h)	*Aspalathus linearis* (Green rooibos)	C2C12 skeletal muscle cells and 3T3-L1 fat cells induced with palmitate.	Aspalathin modulates the major insulin signaling PI3K/AKT and AMPK effectors to ameliorate insulin resistance by increasing glucose transporter expression.	[96]
Bouchardatine (50 mg/kg/days)	*Bouchardatia neurococca*	Male C57BL/6J mice induced with HFD.	Bou may have therapeutic potential for obesity-related metabolic diseases by increasing the capacity of energy expenditure in adipose tissues and liver through a mechanism involving the SIRT1–LKB1–AMPK axis.	[102]
Ginseng oligopeptides (GOPs) (0.125, 0.5 and 2.0 g/kg bw for 7, 24 and 52 weeks)	*Panax ginseng*	Mice that were induced with a high-fat diet for 4 weeks.	Oligopeptides increase the normal content of insulin and protect pancreatic cells from apoptosis associated with type 2 diabetes mellitus by inhibiting NF-κB activity to protect against inflammation due to diabetes.	[103]
Gossypol in vivo (1 and 2.5 mg/kg at 0, 30, 60, 90, 120, 150 and 180 min on glucose tolerance test) and in vitro (25, 50 and 75 μg/mL for 24 h)	*Gossypium* sp.	Mouse myoblast cells (C2C12) and streptozotocin-induced (STZ) mouse myoblasts.	Gossypol (GSP) can activate the insulin receptor substrate 1 (IRS-1)/protein kinase B (Akt) signaling pathway and can translocate glucose transporter 4 (GLUT 4) into the plasma membrane at C2C12 myotube, thereby increasing glucose uptake.	[104]
Hyperoside (200, 100 and 50 mg/kg for 4 weeks)	*Zanthoxylum bungeanum*	Mice that were induced with alloxan and a high-fat diet.	Hyperoside inhibits the phosphorylation of p65/NF-κB, MAPK (including p38, JNK and ERK1/2).	[105]
Isothiocyanate (*Moringa isothiocyanate*/MIC-1) (5 μM for 24 h)	*Moringa oleifera*	HK-2 cells were given high glucose to induce oxidative stress.	Nrf2-ARE is activated by MIC1 to suppress inflammation and reduce oxidative stress.	[99]
Kahweol (2.5 and 5 µM for 24 h)	*Coffea* sp.	INS-1 cells tonal clonal induced with streptozotocin (STZ).	Kahweol downregulates NF-κB, antioxidant proteins, inhibitors of DNA binding and cell differentiation.	[14]
Kukoamine B (50mg/kg/day for 9 weeks)	*Lycium chinense*	Diabetic mouse model (dB/dB) using metabolomics approach (Biocrates p180)	Kukoamine B regulates the NF-κB/PPAR transcriptional pathway to reduce inflammation in diabetes.	[106]
*Lycium barbarum* Polysaccharide (LBPS) (100, 250, and 500mg/kg for 4 weeks)	*Lycium barbarum*	HFD and streptozotocin-induced mice.	It inhibits serum levels of inflammatory factors (IL-2, IL-6, TNF-α, and IFN-α), protects kidney damage and inhibits NF-κB expression.	[107]
Mangiferin (40 mg/kg for 28 days)	*Mangifera indica*	Research on myocardial ischemia-reperfusion (IR) in diabetic rats.	Mangiferin can reduce IR injury in diabetic rats through inhibition of the AGE-RAGE/MAPK pathway thereby preventing oxidative stress, apoptosis and inflammation.	[108]
Morroniside (6.25, 12.5, 25, 50 and 100 μmol/L for 24 h)	*Cornus officinalis* Sieb.	In vitro study using rat renal tubular epithelial cells (mRETCs) induced with palmitate and glucose.	Morroniside increases cholesterol reduction via the PGC1a/LXR pathway and and it also downregulates RAGE, p38MAPK and NF-κB expression via the AGEs/RAGE signaling pathway.	[109]
Nodakenin (NK) (10 and 20 mg/kg for 5 weeks)	*Angelicae gigas*	Male C57BL/6N mice with a high-fat diet.	Administration of NK can improve the phosphorylation level of AMPK, indicating that NK exerts anti-adipogenic and antioxidant effects.	[110]
Onopordopicrin (0.125, 0.25 and 0.5 µg/mL for 24 h)	*Arctium lappa*	A model of human muscle cells exposed to H2O2 oxidative stress.	Onopordopicrin has antioxidant activity by limiting the production of free radicals and DNA damage and through activation of the Nrf2/HO-1 signaling pathway in muscle cells.	[111]
Pectic bee pollen polysaccharide (RBPP-P) in vitro (0.1 mg/mL for 24 h and in vivo (20 mg/kg for 8 weeks)	*Rosa rugosa*	HepG2 cells treated with high-glucose and high-fatty acids and obese mice with a high-fat diet (HFD) inducer.	This polysaccharide is able to decrease hepatic steatosis and insulin resistance by promoting autophagy through AMPK/mTOR-mediated signaling pathways.	[112]
Phanginin A (250 mg/kg for 26 days)	*Caesalpinia sappan*	Male ob/ob mice.	Phanginin A activates SIK1 and causes inhibition of gluconeogenesis with increased PDE4 and inhibition of the cAMP/PKA/CREB pathway in the liver.	[113]
Polyphenol (125–500 mg GP/mL for 8 days)	*Vitis vinivera*	Preadiposit 3T3-F442A cells.	It induces adiposity differentiation through upregulation of GLUT-4, PI3K and adipogenic genes.	[114]
Polysaccharide (200 and 400 mg/kg bw for 8 weeks)	Okra (*Abelmoschus esculentus* (L.) Moench).	Rats that were given a high-fat diet (HFD) combined with injection of 100 mg/kg streptozotocin (STZ) intraperitoneally (ip).	Okra polysaccharide (OP) exert their type 2 antidiabetic effects in part by modulating oxidative stress via Nrf2 transport in the PI3K/AKT/GSK3β pathway.	[72]
Polysaccharide (80, 160 and 320 mg/kg/day for 4 weeks)	*Angelica sinensis*	BALB/C mice induced with a high-fat diet were used.	Angelica sinensis polysaccharide (ASP) is reported to lower blood glucose and improve insulin resistance through regulation of metabolic enzymes and activation of the PI3K/Akt pathway in HFD mice. It can also decrease lipid accumulation and fatty liver by increasing PPARγ expression and activation of the adiponectin signaling pathway SIRT1 and AMPK.	[115]
Polysaccharide (0.1, 1.0, 10 and 100 μg/mL for 0, 12, 24, 48 and 72 h)	*Astragalus mongholicus*	AGE-induced DCM cell model.	Astragalus polysaccharides can decrease intracellular ROS levels, increase SOD activity and GSH-Px and lower MDA and NO levels.	[116]
Procyanidin (25, 50 and 75 μg/mL for 24 h)	*Rubus amabilis*	MIN6 cells were given 0.5 mM palmitate (PA) for 24 h to induce cell apoptosis.	Procyanidin can activate the PI3K/Akt/FoxO1 signal to protect MIN6 cells from apoptosis induced by palmitate induction.	[117]
Puerarin (25, 50 and 100 mg/kg for 12 weeks)	*Pueraria lobata*	Mice induced with streptozotocin.	Puerarin significantly lowers blood sugar levels and prevents cataracts as well as lowers the level of expression of retinal vascular endothelial growth factor and interleukin-1β and increases the expression of Nrf2 and Ho-1 mRNA so that it can reduce oxidative stress in diabetic rats.	[118]
Pyrogallol-phloroglucinol-6,6-bieckol (PPB) (2 mg/kg for 4 weeks)	*Ecklonia cava*	C57BL/6N mice induced with HFD for 8 weeks.	It inhibits RAGE ligands, reduces RAGE expression and binding of RAGE and RAGE ligands and reduces proinflammatory cytokines that cause obesity.	[119]
Resveratrol (1 mg/kg/day for 8 weeks)	*Polygonum cuspidatum*	Goto-Kakizaki (GK) type 2 diabetic female rats.	Resveratrol increases adenine nucleotide and citrate synthase activity by increasing the expression of eNOS-SIRT1 and P-AKT.	[120]
Salidroside (100 mg/kg/day for 5 weeks)	*Rhodiola rosea*	Mice induced by high-fat diet (HFD).	Salidroside suppresses ROS production and inhibits the JNK-caspase apoptotic cascade, inhibiting FOXO-1 by activating AMPK-AKT.	[121]
Saponins (40 mg/kg)	*Momordica carantia* L.	Mice that were induced with a high-fat diet and streptozotocin.	Saponins exhibit hypoglycemic activity possibly via the AMPK/NF-κB signaling pathway by activating AMPK phosphorylation and energy metabolism of the body.	[122]
Simmondsin (10, 20, 40, 80 and 150 µg/mL for simmondsin for 24 h)	*Simmondsia Chinensis*	Fructose-induced oxidative stress in RIN5f beta cells.	Simmondsin is reported to reduce ROS by 69%, activate caspase-3, increase antioxidant defense, inhibit p22phox and increase Nrf2 factor.	[123]
Toosendanin (TSN) in vitro (12.5 nM, 25 and 50 nM for 6 days) in vivo (0.1 mg/kg/day for one month)	*Melia toosendan*	3T3L1 preadipocytes and mice induced with a high-fat diet.	TSN can inhibit adipocyte differentiation and lipid accumulation by activating Wnt/β-catenin signaling, inhibiting mRNA and protein levels of PPAR-γ and C/EBP-α, which proves that TSN can inhibit adipogenesis via its mechanism in inhibiting transcription factor cascades.	[124]

## Data Availability

All data included in the article.
